# Super-antibiofilm effect of N_2_ plasma treated buffer (NPB) against plant pathogenic bacterium

**DOI:** 10.1186/s13036-019-0222-z

**Published:** 2019-12-04

**Authors:** Hyemi Seo, Jisoo Hong, Taeyeol Kim, Won ll Choi, Daekyung Sung, Eunpyo Moon

**Affiliations:** 1 Department of Biological Science, College of Natural Sciences, Ajou University, 206, World cup-ro, Yeongtong-gu, Suwon, 16499 Gyeonggi-do Republic of Korea; 2 Center for Convergence Bioceramic Materials, Korea Institute of Ceramic Engineering and Technology, 202, Osongsaengmyeong 1-ro, Osong-eup, Heungdeok-gu, Cheongju, Chungbuk 28160 Republic of Korea; 3 Microbial Safety Team, National Institute of Agricultural Sciences, Rural Development Administration, 300, Nongsaengmyeong-ro, Deokjin-gu, Jeonju, 54875 Jeonbuk Republic of Korea

**Keywords:** Non-thermal plasma, N_2_ plasma treated buffer, Antibiofilm, Plant pathogenic bacterium, Permeability

## Abstract

Controlling of biofilms formation in numerous pathogenic bacteria is one of the most difficult tasks in the control of bacterial diseases. Plasma has attracted extensive attention due to their potential applications for effective inhibiting of biofilm. Recently, plasma-activated water (PAW) has developed as an alternative method for bacterial inactivation and disinfection of foods owing to advantages of more convenient and efficient storage and transportation than direct plasma application. However, most previous studies about PAW have only focused on the improvement of its antibacterial effect instead of antibiofilm activity. Therefore, we report the development of N_2_ plasma treated buffer (NPB) and the super-antibiofilm effect of NPB against *Pseudomonas syringae* pv. *tomato* DC3000 (*Pst* DC3000) as a plant pathogenic bacterium. Scavenger assays using various antioxidants revealed that reactive oxygen species were involved in the inhibitory cellular actions of NPB, with H_2_O_2_ and singlet oxygen proving essential for bacterial death. Intensive analysis of NPB, stored at different periods and temperatures, showed that the antimicrobial efficacy was well maintained for 3 months at − 80 °C. Importantly, further studies showed that NPB effectively inhibited not only the growth of planktonic *Pst* DC3000 but also biofilm formation. The remarkable inhibition on the biofilm was analyzed and visualized using LIVE/DEAD viability assays and confocal laser scanning microscopy (CLSM) imaging. The 3D CLSM imaging data revealed that the bactericidal activity of NPB was permeable enough to affect the cells embedded inside the biofilm. This prominent permeability could be a crucial feature of NPB contributing to effective super-antibiofilm.

## Introduction

With rapid population growth, the greatest challenge is to produce safe food with high quality considering the new risks encountered during the food production owing to emerging plant-related pathogens [1]. The techniques developed to eliminate plant pathogenic bacteria include the use of pressure, heat, ultraviolet (UV) radiation, ozonation, and more recently, non-thermal atmospheric pressure plasma (NAPP) [2]. Among these, NAPP has been a much-utilized antimicrobial treatment that has the potential to replace the existing treatment, as a promising tool for food preservation. NAPP is more convenient owing to its simplicity and ease of use than the low-pressure cases where more complex control system are needed for decreasing the pressure and combining the plasma with the gas for ionization.

To date, numerous studies regarding the antibacterial properties of NAPP for disinfection of foods have been reported. Many of these studies describe the process on applying the gas plasma directly to get the maximum microbial inactivation efficiency [3–5]. Despite its strong microbial inhibition, there are some negative effects such as small injection area, loss of color, and change in surface topography due to etching and degradation of bioactive compounds [1, 6, 7].

To overcome these problems, recently, plasma-activated water (PAW) was developed as an alternative method for bacterial inactivation and disinfection of foods [8, 9]. Water activated by NAPP creates an acidified solution containing reactive nitrogen and oxygen species, known as PAW, consequently causing oxidative stress in bacterial cells. R. Ma et al. reported the potential of PAW in the inhibition of *S. aureus* inoculated in strawberries [10, 11]. In addition, Y. Xu et al. pointed out that PAW soaking is a promising technique for fresh-keeping of postharvest *A. bisporus* [11, 12]. Previously reports, the main advantages of PAW in plant bacterial inhibition have a lower negative impact on the environment; furthermore, there is no need for storage and transportation of potentially unsafe chemicals. As a safe disinfection material, PAW is a promising alternative to traditional disinfectant applied in the agricultural (sterilization of fruits and vegetables) and food industries (disinfection of poultry products) [11]. However, most previous studies about PAW have only focused on the improvement of its antibacterial effect instead of antibiofilm activity. Generally, biofilms have become problematic in several food industries because biofilm formation on food poses a health risk such as foodborne disease to consumers. In addition, biofilm-related infections result in serious disease that are more resilient to treatment of antimicrobial diseases than infections with free-living bacteria, and thus, effective control of the disease largely depends on the effective control of biofilm formation [13]. For this reason, there is a need for the development of a super-antibiofilm technique to eradicate strong bacteria provided by the extracellular polymeric substance (EPS) and their multilayered structure in biofilm [14, 15].

**Scheme 1:**
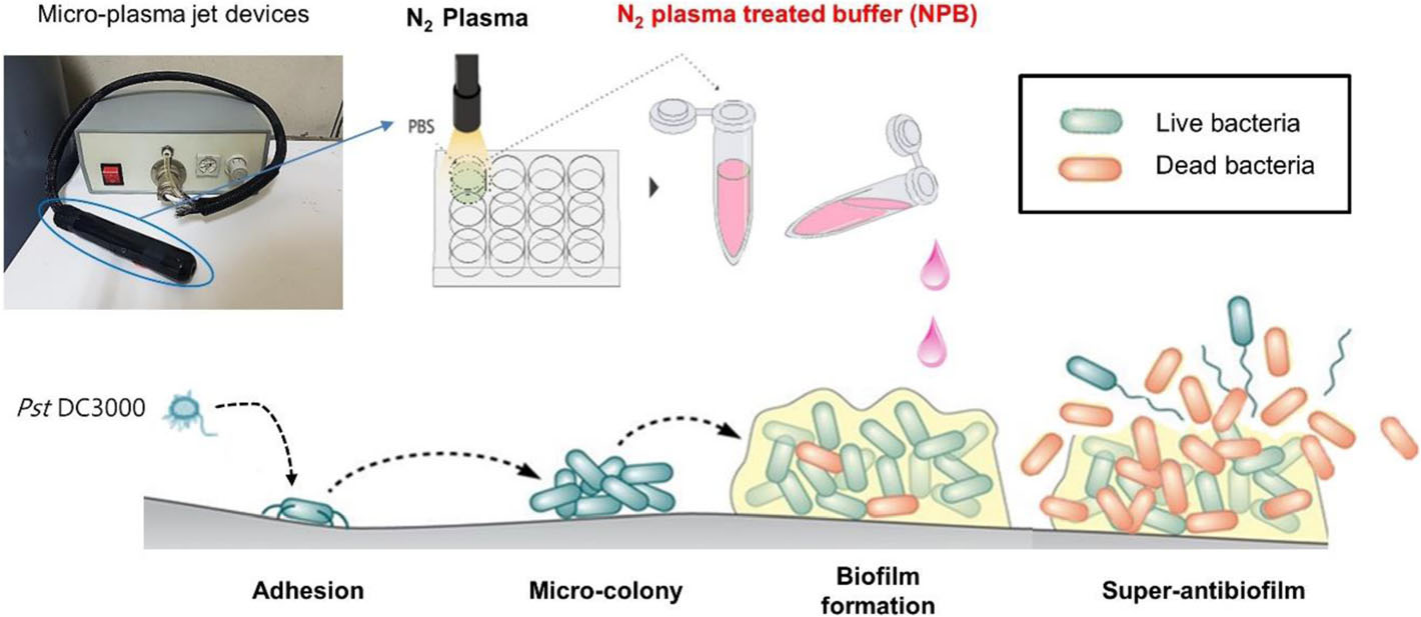
A schematic diagram of micro-plasma jet and the experimental arrangement, including N_2_ plasma treated buffer (NPB) generation, biofilm formation with *Pst* DC3000 as plant pathogenic bacterium and super-antibiofilm effect of NPB

Very recently, we reported the inhibition effects against cancer cells and stimulation effects on tissue regeneration using non-thermal atmospheric pressure plasma generated by micro-jet devices [16–19]. It was demonstrated that reactive nitrogen species (RNS) and reactive oxygen species (ROS) induced by the NAPP effectively enhancing cell death in targeted cancer through the activation of oxidative stress signaling pathways [16]. However, their potential as an antibiofilm inhibitor of plasma treated solution was not examined in the prior report. Therefore, we provide the first report on the development of N_2_ plasma treated buffer (NPB) and the super-antibiofilm effect of NPB against *P. syringae* pv*. tomato* DC3000 (*Pst* DC3000) as a plant pathogen that leads to bacterial speck in tomatoes and other plants (Scheme 1) [20]. *Pst* cells demonstrate adaptive behaviors that increases their resistance to antibiotics through gene expression involved in the formation of biofilms [21]. Here, we have studied the anti-biofilm efficiency of various plasma and discussed the results. Finally, we have provided evidence that the penetration efficacy of NPB against multilayered biofilms is one of the most important properties contributing to its strong antibiofilm activity.

## Materials and methods

### Bacterial strain and biofilm formation

*Pst* DC3000, both planktonic cells and mature biofilms, were treated with plasma. Bacterial strains were grown in Luria-Bertani (LB) media and were shaken until the exponential phase. The strains were then diluted at a concentration of 1:100 in fresh LB medium. The cells grew for 24 h and were harvested by centrifugation at 4000 rpm for 20 min. Cells were re-suspended in PBS (autoclaved, pH 7.2, Sigma Aldrich) and diluted to approximately 108~109 CFU/mL. For mature biofilm development, 1 mL aliquots of diluted bacterial suspension were placed in 12-well PVC plates (SPL life science Co., Pocheon, Korea) with 12 mm Ø microscope cover glasses and incubated statically for 24 h at 37 °C without shaking. Biofilm formation was confirmed by staining with crystal violet [22, 23].

### Micro-plasma jet devices and treatment of plasma

Overall structure of our non-thermal atmospheric-pressure plasma jet micro devices was described previously [16–19]. Plasma device system consisted of a plasma nozzle, a power supply generating an AC voltage of 15 kV at 15 kHz, and a gas supply with a gas flow meter. The structure of the plasma nozzle was similar to the one described previously [18] except that the electrode had 37 holes with a diameter of 400 μm arranged in a honeycomb shape. Discharging for generation of plasma was performed at atmospheric pressure with N_2_, air and helium as the gas sources.

Direct plasma treatment is defined by placing the N_2_ plasma directly onto the bacteria without the medium. Indirect plasma treatment involves N_2_ plasma treatment to bacteria submerged in a PBS solution. To obtain NPB, 1 mL of PBS buffer was placed in a well of a 12-well plate, and then plasma was generated using N_2_ gas with the nozzle positioned at 1 cm above the PBS solution. For the NPB application, 250 μL of NPB was added to an equal volume of PBS solution containing planktonic *Pst* DC3000 cells, or to a formed biofilm, as described in the previous section. Plasmas were applied to *Pst* DC3000 directly in the liquid culture, or indirectly by mixing with PBS pretreated with plasma, and the cells were incubated in the mixture for 20 min at room temperature. After the supernatant was removed, *Pst* DC3000 cells and the biofilm were washed twice with PBS, and then subjected to further analyses.

### LIVE/DEAD bacterial viability assay

The LIVE/DEAD BacLight bacterial viability assay kit was purchased from Invitrogen Co. (Carlsbad, USA). Biofilm grown on 12 mm Ø microscope cover glasses was covered with 300 μL of SYTO9 and PI solution, and then incubated at RT for 15 min in the dark. The cover glass with biofilm was transferred onto a slide glass and observed either under a fluorescence microscope (Zeiss Axioscope 2, Carl Zeiss, Germany) equipped with a GFP and rhodamine filter (X600) or under a confocal laser scanning microscope (CLSM) (Inverted stand, Axio Observer Z1, X630).

### Confocal laser scanning microscopy (CLSM)

The laser was used at 488 nm for excitation, and the emission was observed at 528 nm (SYTO9) and 645 nm (PI) [24]. Zeiss ZEN 2012 software was used to acquire images from nine sections of biofilm, while the Z-stack and CellProfiler software were used to analyze signal intensities and produce 3D images.

### Measurement of ROS

Peroxide solution (H_2_O_2_ in Tris buffer) and luminol/enhancer solution (acridan solution in dioxane and ethanol) were combined in a 40:1 ratio. 50 μL of NPB was mixed with 50 μL of PBS and 50 μL of the peroxide-luminol/enhancer mixture in a 96-well plate. The plate was incubated in the dark for 5 min at RT, and then chemiluminescence was recorded digitally, and the light intensity was measured using a chemi-doc analyzer (Infinity Gel Documentation, Vilber, France).

### Scavenger assay using antioxidants

Various antioxidants were used, including NAC (a scavenger for O free radical), L-histidine (for singlet oxygen), sodium pyruvate (for hydrogen peroxide, H_2_O_2_), mannitol (for hydroxyl radical, HO•), uric acid (for peroxynitrite anion, ONOO^−^), trolox (for peroxyl radical, ROO•) and tiron (for superoxide anion, •O_2_^−^).

*Pst* DC3000 was cultured overnight in LB media and inoculated in fresh LB broth in a 1/100 dilution. After grown for 4 h the cells were harvested by centrifugation at 4000 rpm for 20 min, resuspended in PBS, and then diluted to approximately 10^8^–10^9^ CFU/mL. The cells were treated for 20 min with antioxidants prepared freshly in PBS and sterilized with 0.22 μm filter, at a working concentration of each antioxidant [25]. After the treatment with an antioxidant, NPB was added in equal volume, and then the mixture was allowed to sit for 20 min before serial dilution. 100 μL aliquots of diluted suspension were spread on the surface of LB agar plates, incubated overnight, and then the percentage of surviving cells was calculated by colony counting.

### Statistical analysis

Statistical analyses were performed using SPSS 22.0. The surviving populations of bacteria following the plasma treatments were compared using analysis of variance (ANOVA) [22, 23]. The one-way analysis of variance and Post-Hoc test such as DunnettT3/Scheffe were used to determine whether there were any statistically significant differences between the means of two groups. All experiments were done at least three times, and a *p*-value < 0.05 was considered statistically significant using one-way ANOVA.

## Results and discussion

### Antimicrobial effects of NPB against Pst DC3000

**Fig. 1:**
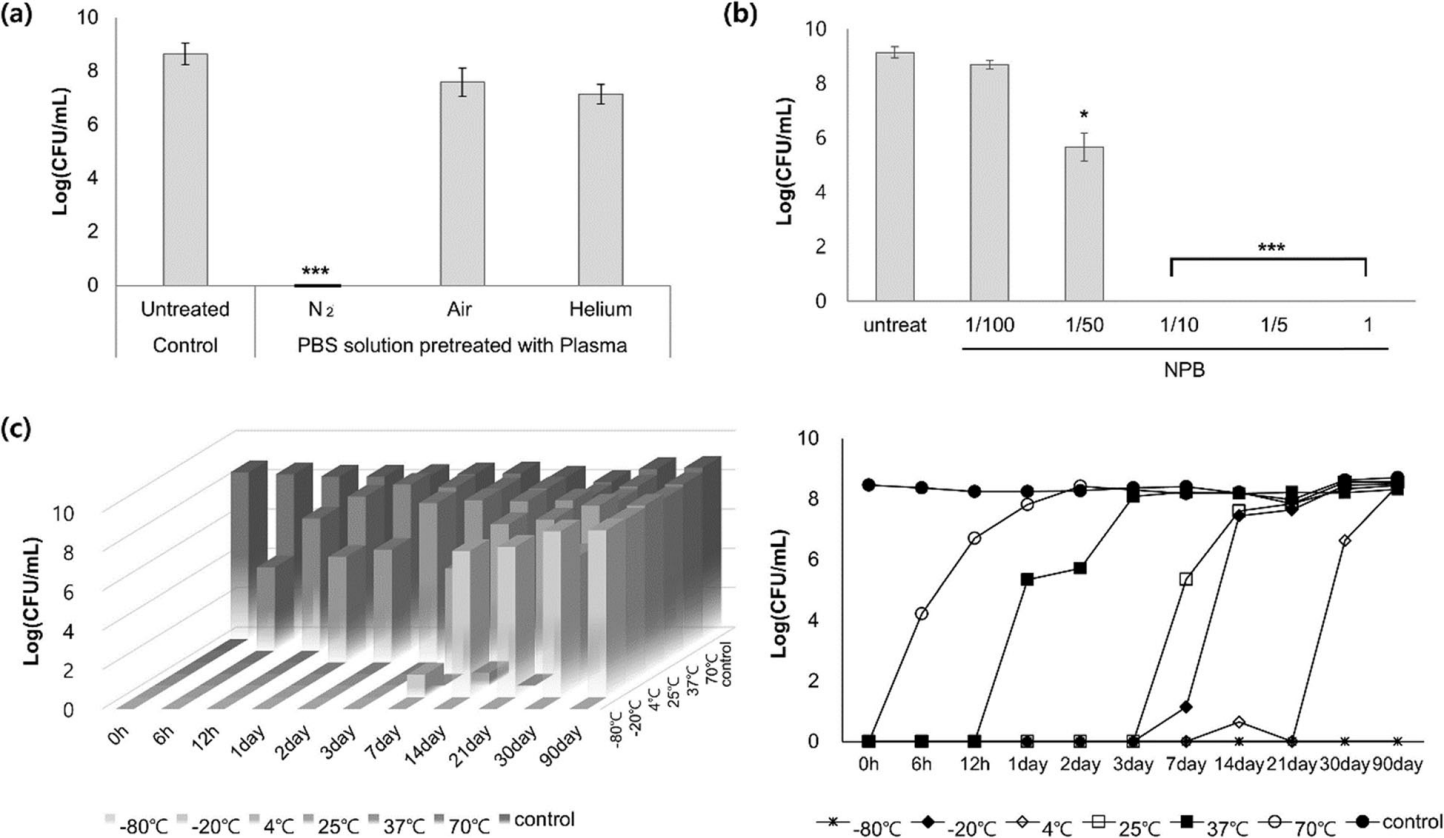
Inhibition efficacy of non-thermal plasma against *Pst* DC3000 under various conditions. The bacteria which survived in the liquid culture from the plasma treatment were grown and counted as the colonies to obtain the value of Log (CFU/mL). (**a**) Inhibition efficacy of plasma according to the gas source: N_2_, air, and helium. (**b**) Inhibition efficacy of NPB according to the concentration (dilution factor) of the plasma. (**c**) Inhibition efficacy of NPB according to the storage time and temperature. The data is visualized in 2 types of graphs. (All experiments were done at least three times. *, *P* < 0.05, **, *P* < 0.01, ***, *P* < 0.001, compared with control)

Plasmas were differently generated using N_2_, air, and helium as the gas source, and PBS solutions, pretreated with various plasmas, were applied against planktonic *Pst* DC3000. When NPB was treated with N_2_ plasma, the resulting plasma had the greatest inhibition of pathogenic bacteria (Fig. 1a). The antimicrobial effect of NPB remained at 100% when it was diluted 10-fold with PBS (Fig. 1b). However, plasmas generated using air and helium showed weak antimicrobial effects. Previous studies by other groups showed that non-thermal atmospheric plasma generally exhibited the highest antimicrobial activity when N_2_ was used, although there were many differences in their plasma devices and in the conditions for their plasma generation [26]. In addition, the studies showed the antimicrobial efficiency of N_2_ plasma was found to be 2–5 log reduction of initial bacterial size [26]. However, the efficiency of N_2_ plasma in this study was shown to be much higher, 8–9 log reduction as shown in Fig. 1. It might be reasonable to speculate that the remarkably high efficiency could be important for the effective inhibition on biofilm discussed in the last two sections.

The inhibition efficacy of NPB stored at different temperatures for different periods of time was examined (Fig. 1c). NPB was stored up to 90 days at six different conditions of temperature, respectively, and administered to *Pst* DC3000 at each time point. The loss of antibacterial activity was estimated by the number of *Pst* DC3000 that had survived after the treatment. The results revealed that the antimicrobial activity was well maintained at − 80 °C at least for 3 months, or at − 4 °C for 3 weeks; the storage condition might be useful for further studies and for the practical application of plasma in agriculture.

### The scavenging effect of various antioxidants on antimicrobial activity of NPB

**Fig. 2:**
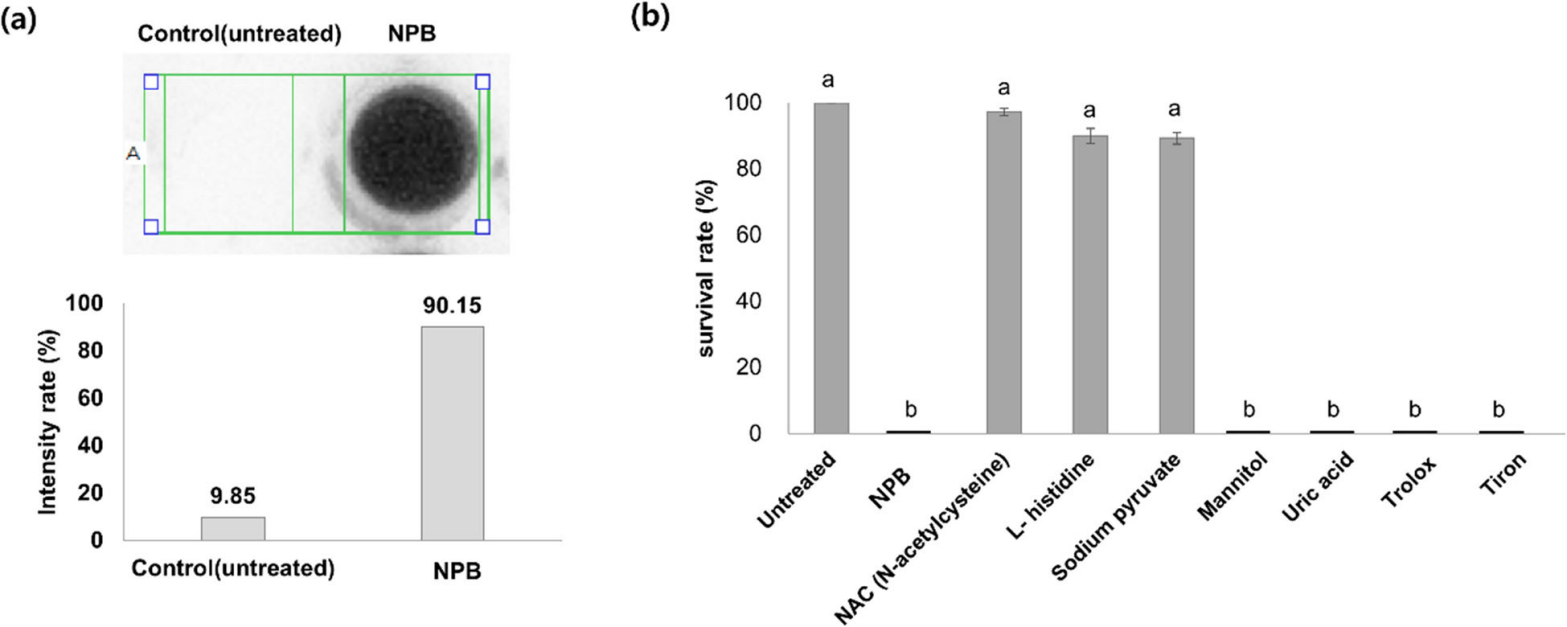
Detection of ROS in NPB, and the scavenging effect of various antioxidants on antimicrobial activity of NPB. (**a**) Concentration of ROS detected in NPB solutions. (**b**) Antioxidants and free radical scavengers with the target ROS shown in parentheses; NAC (O free radical), L-histidine (singlet oxygen), sodium pyruvate (hydrogen peroxide, H_2_O_2_), mannitol (hydroxyl radical, HO•), uric acid (peroxynitrite anion, ONOO^−^), trolox (peroxyl radical, ROO•), and tiron (superoxide anion, •O_2_^−^). Controls: untreated PBS and NPB only. All the experiments were performed at least 3 times

ROS are biologically active molecules that largely contribute to bacterial death in many cases [27]. Thus, we examined the possibility of ROS as the active components in NPB for the inhibitory activity against *Pst* DC3000. When measured using a chemi-doc analyzer, over ten times more ROS was detected in NPB compared to untreated PBS as the control (Fig. 2a). To confirm the possibility of ROS as the active components, NPB scavenging assays using antioxidants were carried out. In this experiment, various antioxidants were used to further distinguish specific ROS responsible for the plasma-mediated bacterial inactivation. As shown in Fig. 2b, NAC, L-histidine, and sodium pyruvate protected *Pst* DC3000 by scavenging ROS in NPB, while mannitol, uric acid, trolox, and tiron were ineffective. Based on the target specificity of L-histidine (a scavenger of singlet oxygen) and sodium pyruvate (a scavenger of hydrogen peroxide), the results revealed that singlet oxygen and hydrogen peroxide were the major active ROS for microbial inhibition against *Pst* DC3000, while other ROS, hydroxyl radical (HO•), peroxynitrite anion (ONOO^−^), peroxyl radical (ROO•), and superoxide anion (•O_2_^−^) were not as effective as the two ROS.

### Inhibitory efficacy of NPB on the biofilm of Pst DC3000

**Fig. 3:**
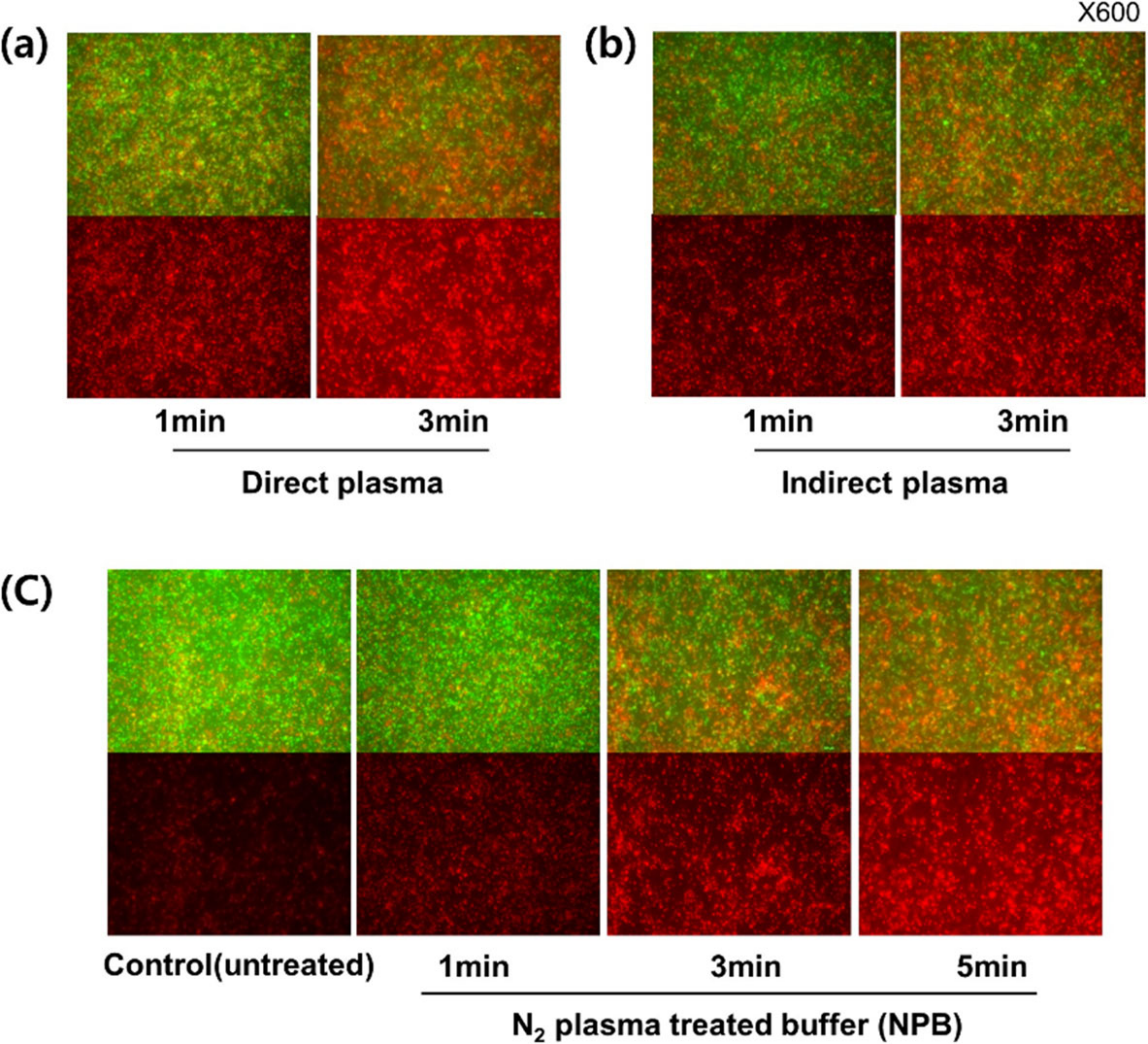
Inhibitory efficacy of N_2_ plasma on biofilm of *Pst* DC3000 according to the treatment time and application methods. The fluorescence microscopy images were obtained after the LIVE/DEAD Bacterial Viability assay on the biofilm treated with plasma. Images in the upper rows are the merge of green (SYTO9, indicating live cells) and red (PI, indicating dead cells), whereas red images in the lower rows are obtained by PI, indicating only dead cells. Application methods used; (**a**) Direct plasma, (**b**) Indirect plasma, (**c**) NPB

*Pst* DC3000 is well known as a biofilm-forming plant pathogenic bacterium, thus effective inhibition on their biofilm is considered as one of the key issues. In addition to the antimicrobial activity against the free-living form of *Pst* DC3000, as discussed in the previous sections, the non-thermal N_2_ plasma (direct and indirect methods) and NPB treatment were evaluated for the inhibition efficacy against their biofilms, depending on the treatment time and application methods, as shown in Fig. 3. Figure 3a and b clearly showed strong inhibition on the target biofilm by N_2_ plasma generated for 3 min, both in direct and indirect applications. Moreover, PBS pretreated for 5 min with N_2_ plasma (NPB) exhibited equally strong antibiofilm activity, as shown in Fig. 3b. In particular, NPB, which had strong anti-biofilm effects, was more convenient and efficient to use and store than N_2_ plasma (both the direct and indirect methods). Thus, the plasma application method using the pretreated plasma solution, NPB, could be of value in future studies.

### 3D analysis on super-antibiofilm efficacy of NPB against Pst DC3000 using CLSM

**Fig. 4:**
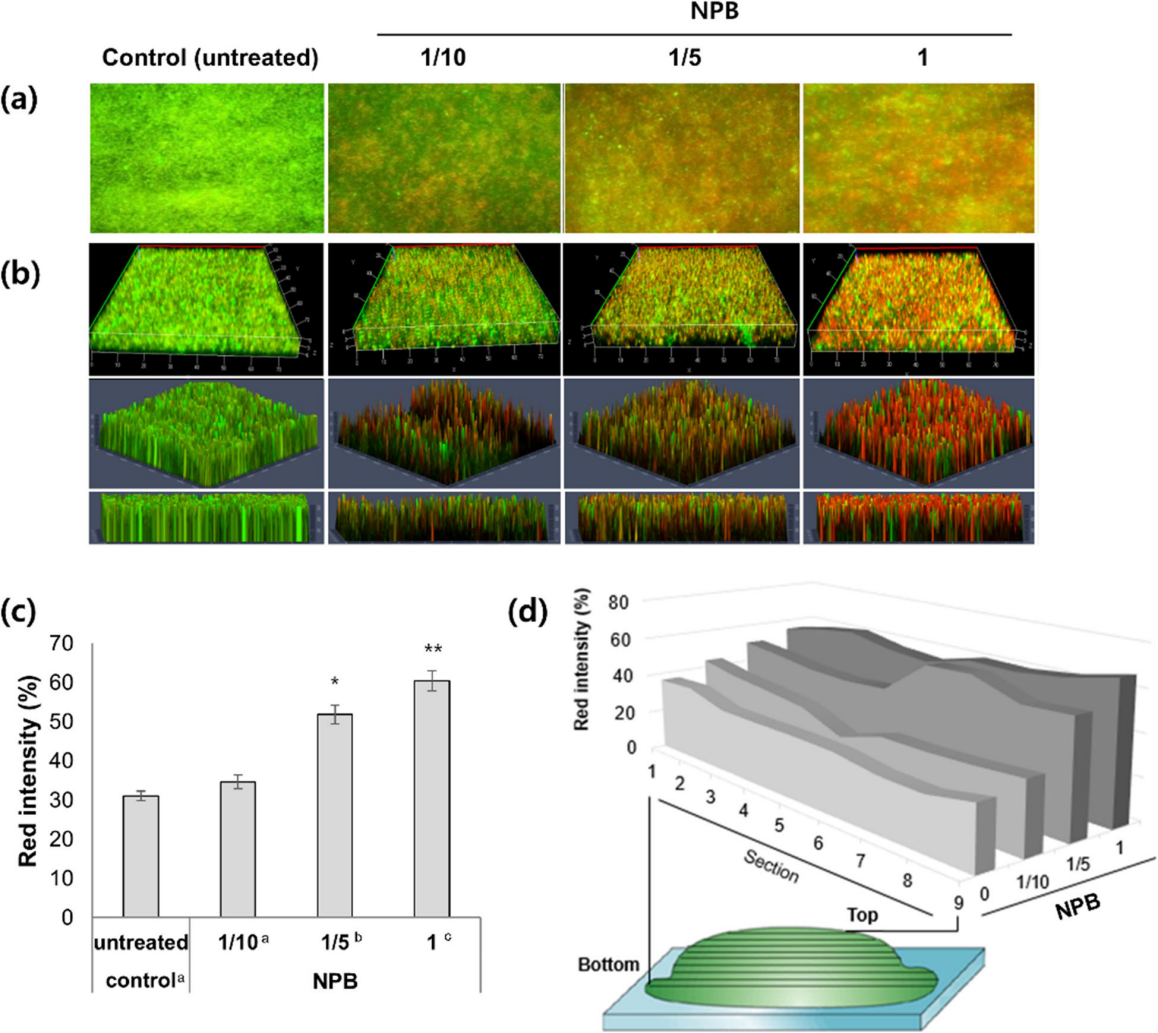
CLSM imaging analysis on biofilms of *Pst* DC3000 after the NPB treatment. All the images were shown as the merge of green and red, as in Fig. 3. The first columns in A and B showed biofilms without plasma treatment, as the control. NPB in three different concentrations (1/10, 1/5, 1) was used in the treatment shown in the next three columns in A and B, respectively. Images of nine sections along the depth of biofilm (sections 9 through 1, from the top, shown in D) were obtained for three different concentrations, respectively. The images were analyzed by the ZEN 2012 program for B, C, and D. (**a**) Fluorescence microscopy images (X600) obtained after the LIVE/DEAD Bacterial Viability assay on the biofilm. (**b**) 3D CLSM images (X630) as Z-stacks of the nine images visualized at three different angles. (**c**) Total red intensity determined from the 3D data sets. (**d**) Red intensity profile as the set of intensity values of sections 1 through 9. All the experiments were repeated at least three times, and statistical significance was determined using one-way ANOVA. (Dunnett T3) (*, P < 0.05; **, P < 0.01, compared with control)

Inhibition efficacy on the biofilm formed by *Pst* DC3000 was further examined using serially diluted NPB. Figure 4 showed fluorescence microscopic 2D images (Fig. 4a) and 3D CLSM images (Fig. 4b) after the LIVE/DEAD Viability assay as the merge of green and red as in Fig. 3. To obtain the final 3D CLSM images in Fig. 4b, each image from nine optical sections, positioned at different depths in the biofilm (shown in Fig. 4d), were acquired as 3D data sets. Z-stacks of nine images were rendered into 3D mode and visualized in three different angles (3 rows in Fig. 4b). The total red intensity of the 3D images was determined using the same program and is shown in Fig. 4c. Antibiofilm activity of NPB was clearly confirmed by red images and total red intensity, which are indicative of dead bacterial cells. Compared to the inhibition efficiency against planktonic *Pst* DC3000, which remained 100% up to a 10-fold dilution of NPB as shown in Fig. 1b, the inhibition activity against the resistant biofilm was slightly less, but well maintained up to a 5-fold dilution, with drastic reduction in a 10-fold dilution (Fig. 4a, b, and c).

It is well known that bacteria embedded inside biofilms survive the inhibitory action of most antibiotic agents because the cells are protected by the barrier created by the biofilm, but the agents effectively inhibit the cells exposed on the surface of the biofilm [28]. To examine the inhibition efficacy of NPB on the inner cells of biofilm, the images from nine optical sections at different depths of biofilm were individually analyzed, and the red intensity of each section was obtained. Figure 4d showed the red intensity profile of the nine sections. Although the red intensity of section 9 (nearest to the surface) was the highest, the intensity of the rest of the sections (down to the deepest section 1) remained the same or slightly less. The 3D analysis revealed NPB effectively inhibited bacterial cells not only near the surface of the biofilm but also embedded deeply inside, suggesting that the inhibitory agent was highly penetrable. It is much more difficult to kill bacteria within biofilm than individual cells, due to the protection provided by the extracellular polymeric substance and their multilayered structure [28]. Thus, the penetration efficacy of NPB against multilayered biofilm should be emphasized and considered as one of the most important features contributing to the efficient antibiofilm activity.

## Conclusion

In this study, antibiofilm activity of the N_2_ plasma treated buffer (NPB) was analyzed using *Pst*. To elucidate the strong antibiofilm efficacy of the NPB, the *Pst* biofilms were optically dissected using CLSM, and the 3D profiling of the inhibition efficiency was elaborately carried out. Inducing the biofilm formation in the culture plates circumvented limitations in the direct analysis with infected plants. The result clearly showed effective penetration of the inhibitory activity through biofilm layers, and thus provided the cellular basis for the super-antibiofilm activity of NPB. The result implies that the high penetration through the coherent multilayers of biofilms could be the most important characteristic of N_2_ plasma that makes it an effective control agent for biofilm-forming plant pathogens. In addition, the storage conditions and treatment methods of NPB provided in this study could be used in practical applications such as decontamination of infected seeds and increased production of crops with less impact on the ecosystem, especially by suppressing biofilm formation.

## Data Availability

The data and materials used and/or analyzed during the current study are available from the corresponding author on reasonable request.
